# Clinical course of asymptomatic malignant pleural effusion in non-small cell lung cancer patients: A multicenter retrospective study

**DOI:** 10.1097/MD.0000000000025748

**Published:** 2021-05-14

**Authors:** Jiyeon Roh, Hyo Yeong Ahn, Insu Kim, Ju Hyeong Son, Hee Yun Seol, Mi Hyun Kim, Min Ki Lee, Jung Seop Eom

**Affiliations:** a Department of Internal Medicine; b Biomedical Research Institute, Pusan National University Hospital; c Department of Thoracic and Cardiovascular Surgery, Pusan National University School of Medicine; d Department of Internal Medicine, Dong-A University Hospital, Busan; e Department of Thoracic and Cardiovascular Surgery; f Department of Internal Medicine, Pusan National University Yangsan Hospital, Yangsan, Republic of Korea.

**Keywords:** chest tubes, computed tomography, lung neoplasms, malignant pleural effusion, non-small cell lung carcinoma

## Abstract

The British Thoracic Society guidelines recommend observation for patients with asymptomatic malignant pleural effusion (MPE). However, asymptomatic MPE can become symptomatic. This study examined the clinical course of asymptomatic MPE in patients with non-small cell lung cancer (NSCLC), including the incidence and timing of symptom development of asymptomatic MPE and the associated factors.

Retrospective data of 4822 NSCLC patients between January 2012 and December 2017 were reviewed. Symptom development of asymptomatic MPE was defined as the development of symptoms requiring additional treatment, such as insertion of a chest tube, within 1 year in patients who lacked MPE symptoms at the time of diagnosis. Clinical information, pathological parameters, and radiological characteristics were reviewed. Patient data up to 1 year from the initial diagnosis were reviewed.

Of 113 patients with asymptomatic MPE, 46 (41%) became symptomatic within 1 year despite appropriate anticancer treatment. The median time to symptom development was 4 months, and 38 patients (83%) developed symptoms within 6 months. Multivariate logistic regression showed that female sex (odds ratio [OR], 0.256; 95% confidence interval [CI], 0.101–0.649; *P* = .004) and the depth of pleural effusion on initial computed tomography (CT) (OR, 0.957; 95% CI, 0.932–0.982; *P* = .001) were independently associated with symptom development of asymptomatic MPE.

A fraction of 41% of patients with asymptomatic MPE became symptomatic within 1 year. Female sex and larger MPE on initial CT were independently associated with symptom development of asymptomatic MPE.

## 1 Introduction

Malignant pleural effusion (MPE) is diagnosed by the detection of malignant cells in pleural fluid or pleural biopsy specimens.^[1]^ Although most malignant diseases can cause MPE, the most common neoplasm involving the pleura is lung carcinoma, accounting for approximately one-third of all MPEs.^[2]^ The median survival of patients with MPE is 3 to 12 months depending on the site of the primary tumor.^[1]^ Lung cancer patients with MPE have the shortest survival with a median of 3 to 4 months.^[3,4]^

The majority of patients with MPE are symptomatic; the most common symptom is dyspnea, which occurs in >50% of patients, followed by cough and chest pain.^[5,6]^ The main objective of MPE treatment is to relieve symptoms and improve quality of life, which is determined by several factors such as symptoms, performance status of the patient, life expectancy, and the type of primary tumor and its response to therapy.^[5,7]^ Management strategies for symptomatic pleural effusion include therapeutic thoracentesis, insertion of indwelling pleural catheter, intercostal tube drainage, and thoracoscopy with chemical pleurodesis.^[8,9]^

According to the British Thoracic Society guidelines, observation is recommended if the patient is asymptomatic and the tumor type is known.^[1]^ However, asymptomatic MPEs become symptomatic in some cases. There is little data about the clinical course of asymptomatic MPE. Here, a multicenter retrospective study was performed to investigate the clinical course of asymptomatic MPE in patients with non-small cell lung cancer (NSCLC), including the incidence and timing of symptom development of asymptomatic MPE as well as the associated factors.

## 2 Materials and methods

### 2.1 Study subjects

In this multicenter retrospective study, clinical, radiologic, and pathologic data were obtained from Pusan National University Hospital, Pusan National University Yangsan Hospital, and Dong-A University Hospital, Republic of Korea. All consecutive patients who were diagnosed histologically as NSCLC between January 1, 2012 and December 31, 2017 were reviewed. NSCLC patients with suspected MPE were included if they met the following criteria: unilateral pleural effusion on the same side of lung cancer^[10]^; age ≥18 years; no cardiomegaly; and no chronic kidney disease and not receiving renal replacement therapy. The exclusion criteria were as follows: patients with minimal pleural effusion, which is defined as a depth of pleural effusion <10 mm measured perpendicular to the parietal pleura on axial computed tomography (CT)^[11,12]^; patients who refused anticancer treatment including the management of MPE; or patients who died within 3 months from initial diagnosis. Patient data up to 1 year from initial diagnosis were reviewed. Because of the observational and retrospective nature of the study, the need for informed consent by the patients was waived. The study was approved by the Institutional Review Board of Pusan National University Hospital (No. 1906-008-080), Pusan National University Yangsan Hospital (No. 05-2019-113), and Dong-A University Hospital (No. DAUHIRB-EXP-20-005).

### 2.2 Data collection

**Figure 1:**
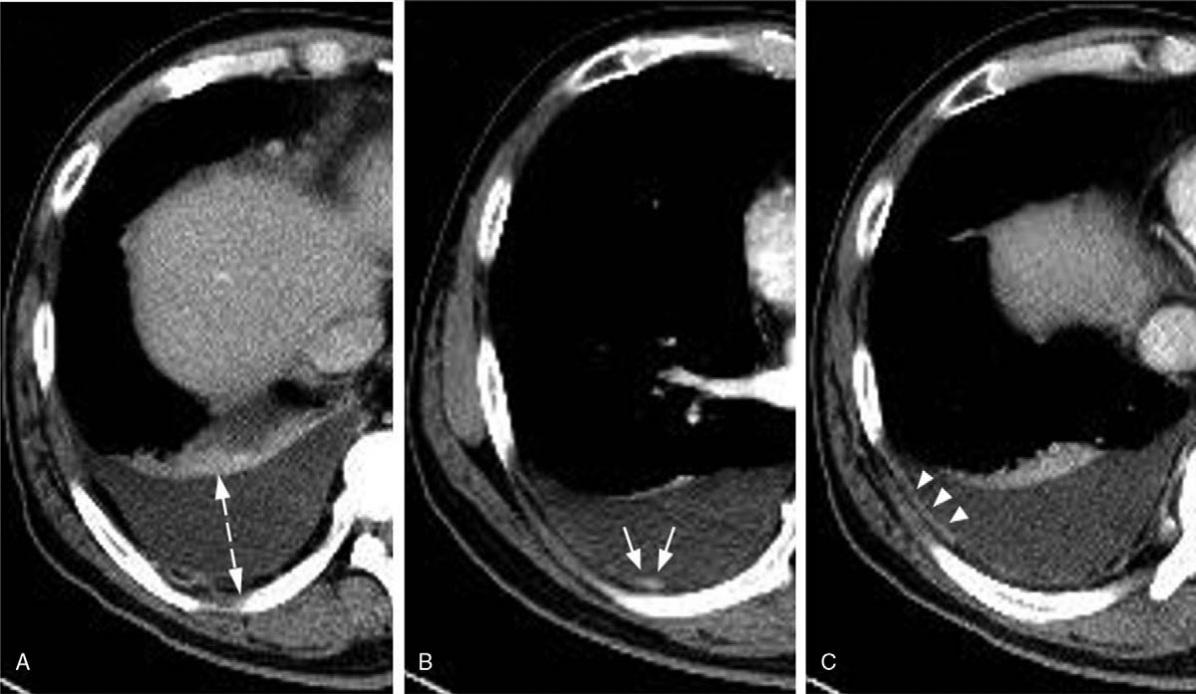
Chest computed tomography (CT) of MPE. (A) The depth of MPE was measured perpendicular to the parietal pleura at the site of greatest depth on axial CT images (dashed arrow). (B) CT image of MPE with pleural nodularity (arrow). (C) Chest CT showing pleural thickening with pleural enhancement (arrowhead). MPE = malignant pleural effusion.

The clinical variables included age, sex, Eastern Cooperative Oncology Group performance status,^[13]^ Charlson Comorbidity Index score,^[14]^ the eighth edition of the TNM classification,^[15]^ number of organs affected by metastasis, initial treatment, and response to first-line anticancer treatment expressed as Response Evaluation Criteria in Solid Tumors (RECIST 1.1).^[16]^ The pathologic variables included histology and gene mutation (epidermal growth factor receptor and anaplastic lymphoma kinase). The date of initial pleural effusion was defined as the date of detection of pleural effusion on chest CT at the time of diagnosis and before the start of lung cancer treatment. The radiologic variables included the depth of pleural effusion, which was measured perpendicular to the parietal pleura on axial CT, and in which the greatest depth was identified by scrolling all the images,^[17]^ loculation of pleural effusion, pleural thickening, and pleural nodularity (Fig. 1).

### 2.3 Classification of patients

Patients were classified into 2 groups as follows: the symptomatic group, which included patients with symptom development of asymptomatic MPE within 1 year, and the asymptomatic group, which included those without symptom development of asymptomatic MPE within 1 year. Patients with symptom development of asymptomatic MPE were defined as patients who had no symptoms related to MPE at the time of diagnosis, but developed symptoms within 1 year from initial diagnosis requiring additional treatment, such as thoracoscopy, insertion of chest tube, or indwelling pleural catheter at the discretion of the physician. Patients were classified into an early symptom development group if conversion of asymptomatic to symptomatic MPE occurred within 3 months, whereas those who developed symptoms after 3 months were classified into the late symptom development group.

### 2.4 Statistical analysis

Results are presented as median (interquartile range [IQR]) for continuous variables and as numbers (percentages) for categorical variables, as appropriate. Categorical variables were compared using Pearson chi-square or Fisher exact test, and Student *t* test or Wilcoxon rank-sum test were used to compare continuous variables. Multivariate logistic regression analyses were performed to identify factors associated with symptom development of asymptomatic MPE with a significance of *P* < .1 in the univariate analyses. *P* < .05 was considered to indicate statistical significance. Receiver operating characteristic curves and the Youden index were used to determine thresholds for continuous variables. Statistical analysis was performed using R statistical program (version 3.6.2, GNU GPL) and SPSS for Windows (version 22.0, SPSS Inc., Chicago, IL).

## 3 Results

### 3.1 Study population

**Figure 2:**
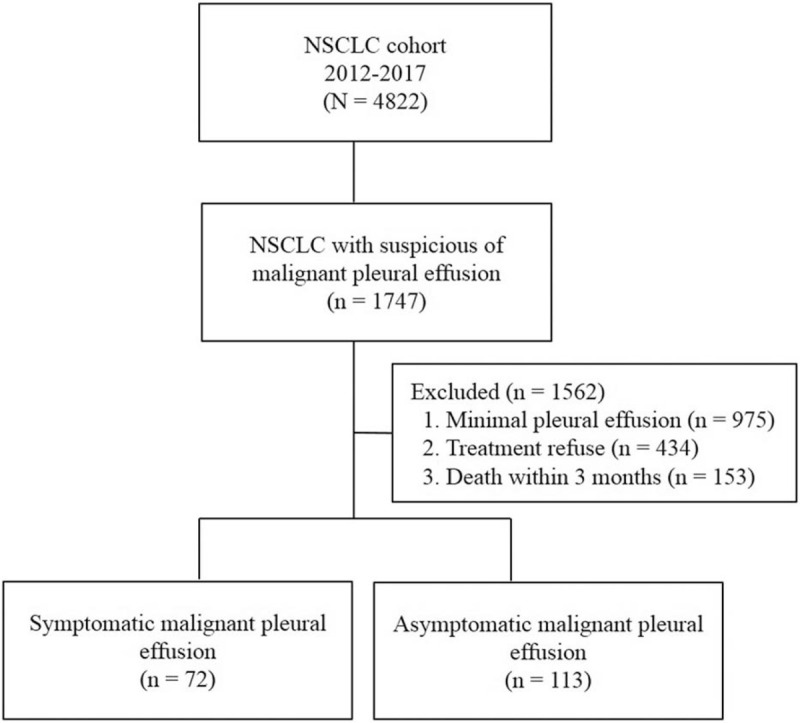
Flow chart of study patients. NSCLC = non-small cell lung cancer.

A cohort of 4822 patients diagnosed as NSCLC during the study period was reviewed (Fig. 2). Suspicion of MPE was found in 1747 patients with NSCLC and 975 (56%) had minimal pleural effusion; 434 (25%) patients who refused anticancer treatment and 153 (9%) who died within 3 months from initial diagnosis were excluded from the analysis. Of 185 NSCLC patients with MPE, 72 (39%) had symptoms related to MPE at the time of diagnosis and were managed with intercostal tube drainage or thoracoscopy before the start of first-line treatment. Finally, 113 patients (61%) with asymptomatic MPE who received anticancer treatment and survived >3 months were included in the study. The baseline characteristics of 113 study patients are shown in Table 1. The median age was 67 years and 33% were women. Eighty-five patients (75%) were diagnosed as adenocarcinoma, and driver mutations were detected in 42 (37%) patients.

**Table 1:** Baseline characteristics of 113 study patients with asymptomatic malignant pleural effusion.

Variables	Median (IQR) or No. (%)
Age, y	67 (59–74)
Female gender	37 (33)
ECOG performance status	
≤1	87 (77)
>1	26 (23)
Charlson Comorbidity Index score	
≤1	19 (17)
>1	94 (83)
T stage	
≤2	53 (47)
>2	60 (53)
N stage	
≤1	28 (25)
>1	85 (75)
M stage	
1a	37 (33)
1b	10 (9)
1c	66 (58)
Number of metastatic organs involved	
≤2	61 (54)
>3	52 (46)
Histology	
Adenocarcinoma	85 (75)
Squamous cell carcinoma	24 (21)
Others	4 (4)
Driver mutation detected^∗^	42 (37)
Targeted agent as first-line treatment	32 (28)
Response to first-line treatment^†^	
Partial response	20 (18)
Stable disease or progressive disease	93 (82)

ECOG = Eastern Cooperative Oncology Group, IQR = interquartile range.

∗ Epidermal growth factor receptor mutation or anaplastic lymphoma kinase rearrangement.

† Treatment responses are expressed according to Response Evaluation Criteria in Solid Tumor (RECIST 1.1).

### 3.2 Clinical course of asymptomatic MPE

**Figure 3:**
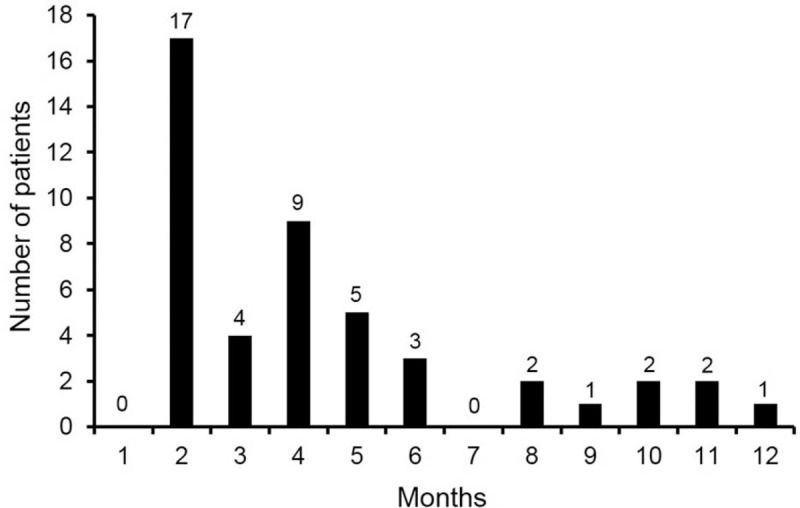
Time to symptom development of asymptomatic malignant pleural effusion within 1 year.

Of the 113 NSCLC patients with asymptomatic MPE, 46 (41%) developed symptomatic disease within 1 year. Figure 3 shows the time from initial diagnosis to symptom development of asymptomatic MPE. The number of patients who developed symptomatic disease within 6 months was 38 (83%), and the median time to symptom development was 4 months (IQR, 2–5). Of all patients with asymptomatic MPE, 24 (30%) died within 1 year and 13 (38%) developed symptomatic disease before death; MPE-related symptoms developed within 6 months in all 13 patients, and the median time to symptom development was 4 months (IQR, 2–5) (see Figure, Supplemental Figure 1, which shows the time to development of symptomatic disease in 13 NSCLC patients with asymptomatic MPE who died within 1 year).

Of 46 patients with symptom development of asymptomatic MPE, 21 (46%) were classified into the early symptom development group and 25 (54%) into the late symptom development group (see Table, Supplemental Table 1, which shows the comparison according to the time of symptom development). The depth of pleural effusion on initial CT scan was significantly larger in the early symptom development group than in the late symptom development group (36 mm vs 23 mm, *P* = .008). There was no significant difference in mortality within 1 year between the early and late symptom development groups (29% vs 28%, *P* = .966).

### 3.3 Factors associated with symptom development of asymptomatic MPE

The clinical characteristics, histologic profile, and radiologic variables of the symptomatic and asymptomatic groups are shown in Table 2. The symptomatic group was older (71 years vs 66 years, *P* = .008), had a higher proportion of women (46% vs 24%, *P* = .015), and had a greater depth of pleural effusion on CT scan (28 mm vs 19 mm, *P* = .010) than the asymptomatic group. The proportion of patients with a high Charlson Comorbidity Index score (91% vs 78%, *P* = .056) and that of patients with pleural nodularity (39% vs 22%, *P* = .054) were higher in the symptomatic group than in the asymptomatic group, although the differences did not reach statistical significance.

**Table 2:** Comparisons between symptomatic and asymptomatic groups.

Variables	Symptomatic group (n = 46)	Asymptomatic group (n = 67)	*P-*value
Age, y	71 (64–75)	66 (58–73)	.008
Female gender	21 (46)	16 (24)	.015
ECOG performance status			
≤1	39 (85)	48 (72)	.103
>1	7 (15)	19 (28)	
Charlson Comorbidity Index score			
≤1	4 (9)	15 (22)	.056
>1	42 (91)	52 (78)	
T stage			
≤2	20 (43)	33 (49)	.546
>2	26 (57)	34 (51)	
N stage			
≤1	9 (19)	19 (28)	.287
>1	37 (44)	48 (72)	
M stage			
1a	17 (37)	20 (30)	.234
1b	6 (13)	4 (6)	
1c	23 (50)	43 (64)	
Histology			
Squamous cell carcinoma	10 (22)	14 (21)	.914
Non-squamous cell carcinoma	36 (78)	53 (79)	
Driver mutation detected^∗^	13 (28)	29 (43)	.104
Targeted agent as first-line treatment	11 (24)	21 (31)	.389
Median depth of MPE on CT, mm	28 (17–44)	19 (14–30)	.010
Pleural thickening on CT	14 (30)	13 (19)	.177
Pleural nodularity on CT	18 (39)	15 (22)	.054
Loculation of MPE on CT	0 (0)	4 (5)	.146

CT = computed tomography, ECOG = Eastern Cooperative Oncology Group, MPE = malignant pleural effusion.

∗ Epidermal growth factor receptor mutation or anaplastic large-cell lymphoma kinase rearrangement.

Variables with a significance of *P* < .1 in the univariate analysis were subjected to multivariate logistic regression analysis to verify the independent factors associated with symptom development of asymptomatic MPE (Table 3). Female sex (odds ratio [OR], 0.256; 95% confidence interval [CI], 0.101–0.649; *P* = .004) and depth of pleural effusion on CT (OR, 0.965; 95% CI, 0.932–0.982; *P* = .001) were independently associated with symptom development of asymptomatic MPE in NSCLC patients. The area under the receiver operating characteristic curve for optimal thresholds for depth of MPE to predict symptom development was 0.642 (95% CI, 0.546–0.730). The optimal cutoff value of depth of MPE was 24.5 mm, which was obtained from the maximum Youden index (sensitivity, 65.7%; specificity, 59.7%) (see Figure, Supplemental Figure 2, which shows the area under the receiver operating characteristic curve for optimal thresholds for depth of malignant pleural effusion).

**Table 3:** Multivariate logistic regression analysis to identify factors associated with conversion of asymptomatic malignant pleural effusion into symptomatic malignant pleural effusion.

Variables	Odds ratio (95% confidence interval)	*P*-value
Age (per year)	0.445 (0.158–1.257)	.127
Female gender	0.256 (0.101–0.649)	.004
Charlson Comorbidity Index score	0.354 (0.070–1.779)	.208
Depth of MPE on CT, mm	0.957 (0.932–0.982)	.001
Pleural nodularity on CT	0.510 (0.198–1.312)	.163

CT = computed tomography, MPE = malignant pleural effusion.

## 4 Discussion

This multicenter retrospective study investigated the clinical course of asymptomatic MPE in NSCLC patients. Conversion of asymptomatic MPE into symptomatic MPE occurred in 41% of patients within 1 year, and the median time to the development of symptomatic disease was 4 months. Most patients developed symptoms related to MPE within 6 months despite the absence of symptoms at diagnosis. Patients with severe MPE tended to have symptom development within 3 months. To the best of our knowledge, the present study is the first to investigate the clinical course of asymptomatic MPE in NSCLC patients.

The present results indicate that women with asymptomatic MPE have a significantly higher risk of developing symptoms than men. A previous study showed that the absolute lung volume and its potential space of pleural cavity are correlated with height.^[18]^ Women are generally shorter than men, suggesting that women have a relatively small pleural space and lung volume, which are related to the manifestation of MPE symptoms. For this reason, breathlessness is higher in women than in men in the general population (12% vs 6%, respectively).^[18]^

Little is known about the impact of the amount of MPE. Although the presence of MPE is considered a poor prognostic factor, Abrao et al^[19]^ showed that the volume of MPE is not correlated with survival. A large pleural effusion is considered important evidence of MPE itself in the differential diagnosis. Alemán et al^[20]^ demonstrated that in patients with no malignant cells in the cytology specimen of pleural effusion, the depth of pleural effusion on CT is positively associated with the probability of MPE. We found that the initial volume of MPE is an important factor predicting symptom development of asymptomatic MPE within 1 year. These findings indicate that measuring MPE depth on CT, which can be performed by scrolling down computed tomography images, may predict symptom development in patients with asymptomatic MPE.^[17]^

According to the American Thoracic Society guidelines, pleural drainage in patients with asymptomatic MPE is recommended under certain conditions, such as to define cancer stage or obtain molecular markers.^[21]^ However, delayed intervention after the conversion of asymptomatic into symptomatic MPE could lead to septation or loculation of MPE.^[21–23]^ In addition, trapped lung syndrome develops in up to 10% to 30% of patients when MPE is left untreated, and in these cases, complete lung re-expansion can be challenging.^[22]^ The present findings suggest that in certain clinical situations, such as in female patients and in those with large MPE (effusion depth >24.5 mm on axial CT scan), a more aggressive and earlier management of MPE is necessary despite the presence of asymptomatic disease.

The present study had several limitations. First, because diagnostic thoracentesis was not possible in all NSCLC patients, suspicion of MPE was defined as unilateral pleural effusion ipsilateral to the primary lung tumor. To minimize the contamination of non-malignant effusion in the study population, we inevitably excluded patients with minimal pleural effusion, because it is difficult to determine whether it is malignant based on CT scan only. In addition, we excluded cases of pleural effusion with cardiomegaly, which is defined as a cardiothoracic ratio (cardiac diameter divided by thoracic diameter measured on postero-anterior chest radiography) ≥0.5, and pleural effusion in patients with chronic kidney diseases or those undergoing renal replacement therapy.^[24,25]^ Therefore, the potential for selection bias in the patient population cannot be excluded. Second, because a pulmonary function test is not mandatory for stage IV lung cancer patients, discriminating symptoms related to the underlying lung function was challenging. Third, at the time of data collection, testing for c-ros oncogene 1 mutation was not common practice in the Republic of Korea because of insurance issues. In addition, immunotherapy was not a treatment option during the study period; therefore, the results do not reflect the recent trends in lung cancer treatment. Lastly, the relatively small sample size may have limited the interpretation of the results. Further prospective studies including larger cohorts are needed to verify the present results.

In conclusion, investigation of the clinical course of asymptomatic MPE in NSCLC patients who survived >3 months showed that 41% of asymptomatic MPE patients eventually develop symptoms within 1 year. Female sex and the initial amount of MPE were identified as independent predictors of symptom development of asymptomatic MPE in NSCLC patients. These data may be of value for decision-making regarding early and aggressive management of MPE.

## Author contributions

**Conceptualization:** Min Ki Lee, Jung Seop Eom.

**Data curation:** Hyo Yeong Ahn.

**Formal analysis:** Jiyeon Roh.

**Funding acquisition:** Insu Kim.

**Investigation:** Insu Kim, Ju Hyeong Son.

**Methodology:** Ju Hyeong Son.

**Project administration:** Hee Yun Seol.

**Resources:** Hee Yun Seol.

**Software:** Ju Hyeong Son, Mi Hyun Kim.

**Supervision:** Jung Seop Eom.

**Validation:** Hyo Yeong Ahn, Mi Hyun Kim.

**Visualization:** Min Ki Lee.

**Writing – original draft:** Jiyeon Roh.

**Writing – review & editing:** Jung Seop Eom.

## Supplementary Material

Supplemental Digital Content

## Supplementary Material

Supplemental Digital Content

## Supplementary Material

Supplemental Digital Content
