# Supplementary material for: Clinical course of asymptomatic malignant pleural effusion in non-small cell lung cancer patients: A multicenter retrospective study
Source: Medicine (Baltimore). 2021 May 14;100(19):e25748. doi: 10.1097/MD.0000000000025748 (PMC8133234; doi:10.1097/MD.0000000000025748)

**Supplemental files legends**

Supplemental Figure 1. Time to development of symptomatic disease in 13 NSCLC patients with asymptomatic MPE who died within 1 year.


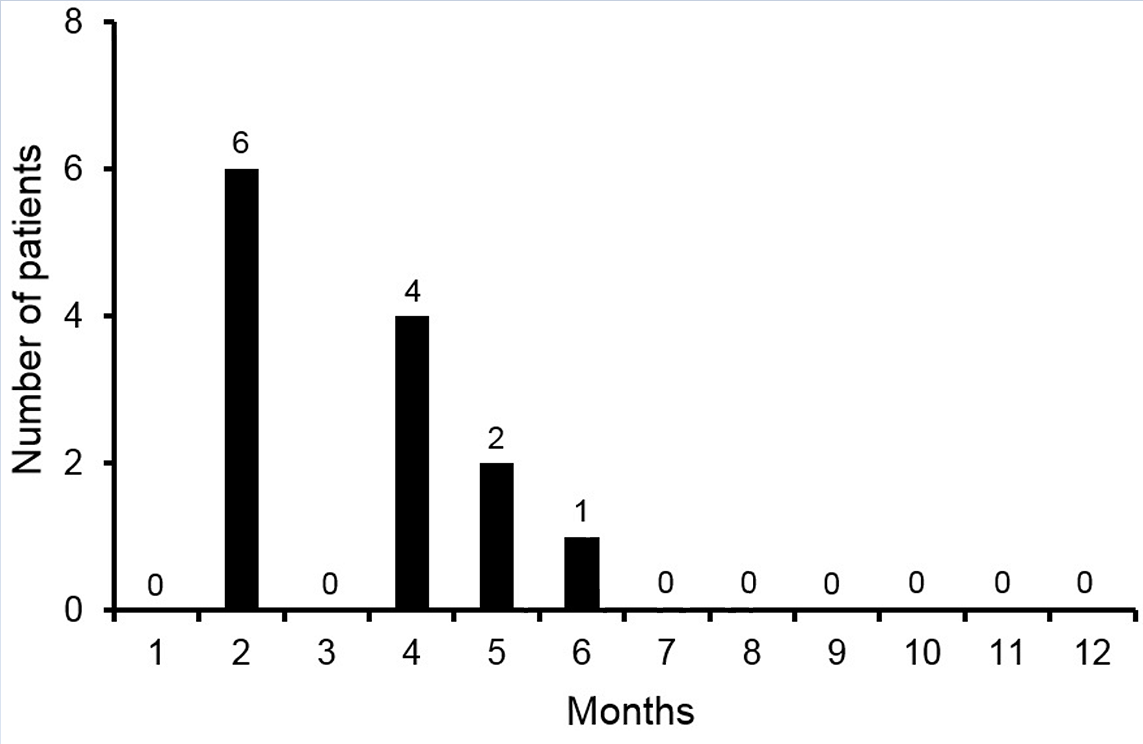

Supplement: Supplemental Digital Content [file medi-100-e25748-s001.docx]
