# Supplementary material for: Clinical course of asymptomatic malignant pleural effusion in non-small cell lung cancer patients: A multicenter retrospective study
Source: Medicine (Baltimore). 2021 May 14;100(19):e25748. doi: 10.1097/MD.0000000000025748 (PMC8133234; doi:10.1097/MD.0000000000025748)
Supplement: Supplemental Digital Content [file medi-100-e25748-s002.docx]

Supplemental Table 1. Comparisons according to the time of symptomatic change.

| Variables | Early symptomatic change group  (n = 21) | Late symptomatic change group  (n = 25) | *P*-value |
| --- | --- | --- | --- |
| Age, years | 67 (62–76) | 71 (67–75) | 0.305 |
| Female gender | 7 (33) | 14 (56) | 0.124 |
| ECOG performance status |  |  |  |
| ≤ 1 | 17 (81) | 22 (88) | 0.507 |
| > 1 | 4 (19) | 3 (12) |  |
| Charlson Comorbidity Index score |  |  |  |
| ≤ 1 | 3 (14) | 1 (4) | 0.217 |
| > 1 | 18 (86) | 24 (96) |  |
| T stage |  |  |  |
| ≤ 2 | 9 (43) | 11 (44) | 0.938 |
| > 2 | 12 (57) | 14 (56) |  |
| N stage |  |  |  |
| ≤ 1 | 5 (24) | 4 (16) | 0.506 |
| > 1 | 16 (76) | 21 (84) |  |
| M stage |  |  |  |
| 1a | 8 (38) | 9 (36) | 0.809 |
| 1b | 2 (10) | 4 (16) |  |
| 1c | 11 (52) | 12 (48) |  |
| Histology |  |  |  |
| Adenocarcinoma | 16 (76) | 19 (76) | 0.517 |
| Squamous cell carcinoma | 4 (19) | 6 (24) |  |
| Others | 1 (5) | 0 (0) |  |
| Driver mutation detected* | 8 (38) | 5 (20) | 0.175 |
| Targeted agent as first-line treatment | 6 (29) | 5 (20) | 0.497 |
| Median depth of MPE on CT, mm | 36 (23–54) | 23 (16–31) | 0.008 |
| Pleural thickening on CT | 7 (33) | 7 (28) | 0.695 |
| Pleural nodularity on CT | 10 (48) | 8 (32) | 0.280 |
| Loculation of MPE on CT | 0 (0) | 0 (0) |  |
| Death within 1 year | 6 (29) | 7 (28) | 0.966 |

ECOG = Eastern Cooperative Oncology Group, MPE = malignant pleural effusion.

* Epidermal growth factor receptor mutation or anaplastic lymphoma kinase rearrangement.
