# Supplementary material for: Clinical course of asymptomatic malignant pleural effusion in non-small cell lung cancer patients: A multicenter retrospective study
Source: Medicine (Baltimore). 2021 May 14;100(19):e25748. doi: 10.1097/MD.0000000000025748 (PMC8133234; doi:10.1097/MD.0000000000025748)

**Supplemental files legends**

Supplemental Figure 2. The area under the receiver operating characteristic curve for optimal thresholds for depth of malignant pleural effusion.


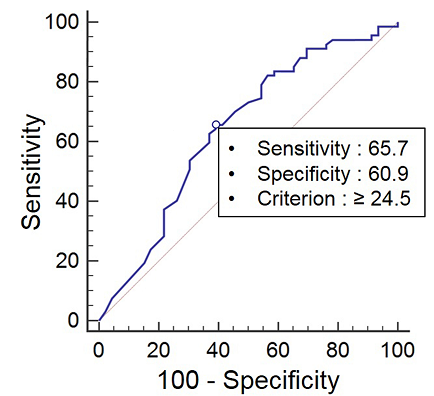

Supplement: Supplemental Digital Content [file medi-100-e25748-s003.docx]
